# β-Cyanoalanine synthase protects mites against Arabidopsis defenses

**DOI:** 10.1093/plphys/kiac147

**Published:** 2022-03-28

**Authors:** Sameer Dixit, Emilie Widemann, Nicolas Bensoussan, Golnaz Salehipourshirazi, Kristie Bruinsma, Maja Milojevic, Akanchha Shukla, Luis C Romero, Vladimir Zhurov, Mark A Bernards, Maksymilian Chruszcz, Miodrag Grbić, Vojislava Grbić

**Affiliations:** Department of Biology, The University of Western Ontario, London, Ontario, Canada N6A 5B7; Department of Biology, The University of Western Ontario, London, Ontario, Canada N6A 5B7; Department of Biology, The University of Western Ontario, London, Ontario, Canada N6A 5B7; Department of Biology, The University of Western Ontario, London, Ontario, Canada N6A 5B7; Department of Biology, The University of Western Ontario, London, Ontario, Canada N6A 5B7; Department of Biology, The University of Western Ontario, London, Ontario, Canada N6A 5B7; Department of Biology, The University of Western Ontario, London, Ontario, Canada N6A 5B7; Instituto de Bioquímica Vegetal y Fotosíntesis, Consejo Superior de Investigaciones Científicas and Universidad de Sevilla, E-41092 Seville, Spain; Department of Biology, The University of Western Ontario, London, Ontario, Canada N6A 5B7; Department of Biology, The University of Western Ontario, London, Ontario, Canada N6A 5B7; Department of Chemistry and Biochemistry, University of South Carolina, Columbia, South Carolina, 29208, USA; Department of Biology, The University of Western Ontario, London, Ontario, Canada N6A 5B7; Department of Biology, The University of Western Ontario, London, Ontario, Canada N6A 5B7; Instituto de Ciencias de la Vid y del Vino, 26006 Logroño, Spain

## Abstract

Glucosinolates are antiherbivory chemical defense compounds in Arabidopsis (*Arabidopsis thaliana*). Specialist herbivores that feed on brassicaceous plants have evolved various mechanisms aimed at preventing the formation of toxic isothiocyanates. In contrast, generalist herbivores typically detoxify isothiocyanates through glutathione conjugation upon exposure. Here, we examined the response of an extreme generalist herbivore, the two-spotted spider mite *Tetranychus urticae* (Koch), to indole glucosinolates. *Tetranychus urticae* is a composite generalist whose individual populations have a restricted host range but have an ability to rapidly adapt to initially unfavorable plant hosts. Through comparative transcriptomic analysis of mite populations that have differential susceptibilities to Arabidopsis defenses, we identified *β-cyanoalanine synthase* of *T. urticae* (*TuCAS*), which encodes an enzyme with dual cysteine and β-cyanoalanine synthase activities. We combined Arabidopsis genetics, chemical complementation and mite reverse genetics to show that *TuCAS* is required for mite adaptation to Arabidopsis through its β-cyanoalanine synthase activity. Consistent with the β-cyanoalanine synthase role in detoxification of hydrogen cyanide (HCN), we discovered that upon mite herbivory, Arabidopsis plants release HCN. We further demonstrated that indole glucosinolates are sufficient for cyanide formation. Overall, our study uncovered Arabidopsis defenses that rely on indole glucosinolate-dependent cyanide for protection against mite herbivory. In response, Arabidopsis-adapted mites utilize the β-cyanoalanine synthase activity of TuCAS to counter cyanide toxicity, highlighting the mite’s ability to activate resistant traits that enable this extreme polyphagous herbivore to exploit cyanogenic host plants.

## Teaser

Indole glucosinolate-dependent cyanides deter mite herbivory on Arabidopsis plants; in response, mites utilize β-cyanoalanine synthase activity to counter cyanide toxicity.

## Introduction

The arms race between plants and herbivores, occurring over millions of years, has led to reiterative evolution and diversification of adaptive traits in both host plants and herbivores (Wheat et al., 2007). Specialist herbivores evolved a variety of highly efficient resistance traits against a narrow range of plant host defenses they encounter (Despres et al., 2007; Heidel-Fischer and Vogel, 2015). Generalist herbivores, on the other hand, evolved an innate ability to feed on a wide range of hosts. Generalists use two main strategies to overcome plant host defenses. Broad-generalists, whose individuals have the ability to feed on the whole range of species’ hosts, are assumed to rely on transcriptional plasticity of genes encoding effector proteins and/or detoxification enzymes leading to the attenuation of plant defenses and to increased detoxification potential, respectively (Li et al., 2007; Hogenhout and Bos, 2011; Kaloshian and Walling, 2016). However, resistance traits used by composite generalist herbivores, regarded as a sum of populations that themselves thrive on a subset of potential hosts, are not well understood (Fox and Morrow, 1981; Peccoud et al., 2009; Barrett and Heil, 2012).

Cyanogenesis is a broadly distributed chemical defense that is reported in over 2,500 plant species (Poulton, 1990). Cyanogenic glucosides are the most common cyanogenic compounds that are synthesized as inactive precursors and require modification by β-glucosidases and α-hydroxynitrile lyases for hydrogen cyanide (HCN) release (Conn, 1981; Frehner and Conn, 1987; Selmar et al., 1987, 1989; Poulton, 1990; Møller and Seigler, 1999; Zagrobelny et al., 2004). Cyanide is a potent inhibitor of mitochondrial oxidative phosphorylation (Isom and Way, 1984) and is an effective deterrent against herbivory (Tattersall et al., 2001; Hay-Roe et al., 2011; Wybouw et al., 2012, 2014). Herbivores that can feed on cyanogenic plants overcome cyanide toxicity mainly through sequestration of ingested cyanogenic glucosides and modulation of feeding behavior that minimizes the uptake of cyanogenic compounds (Zagrobelny et al., 2008; Pentzold et al., 2014; Zagrobelny et al., 2014). In addition, some arthropods have the ability to detoxify cyanide. For example, the whitefly *Bemisia tabaci* modifies cyanogenic glucosides so that they cannot be activated by plant enzymes (Easson et al., 2021), while lepidopteran insects and mites have the ability to detoxify cyanide by enzyme that is encoded by the *β-cyanoalanine synthase* (*CAS*) gene (Meyers and Ahmad, 1991; Stauber et al., 2012; Wybouw et al., 2014).

Arabidopsis (*Arabidopsis thaliana*) has been a useful plant model to study plant–herbivore interactions. Glucosinolates, amino acid-derived secondary metabolites, are considered to be the major antiherbivory chemical defense compounds in Arabidopsis. Among this class of secondary metabolites, aliphatic glucosinolates (derived from methionine) and indole glucosinolates (derived from tryptophan [Trp]) are the most abundant in Arabidopsis (Wittstock and Halkier, 2002; Halkier and Gershenzon, 2006). Variations in the side-chain length and side-chain modifications, combined with the hydrolysis and conjugation of glucosinolate breakdown products with various adducts, contribute to the structural diversity of glucosinolate-derived compounds (Rask et al., 2000; Kliebenstein et al., 2005). Among them, the isothiocyanates have the greatest toxicity to a wide range of herbivores (Wittstock et al., 2003). Brassicaceae-specialized herbivores have evolved various strategies to avoid isothiocyanate toxicity and develop glucosinolate resistance. For example, lepidopteran specialists *Plutella xylostella* and *Pieris rapae* express enzymes in the larval gut that modify glucosinolates and prevent their hydrolysis, or redirect the synthesis of isothiocyanates toward nitriles that are less toxic compounds, respectively (Agerbirk et al., 1998; Ratzka et al., 2002; Wittstock et al., 2004). While nitriles derived from the aliphatic glucosinolates are directly excreted in the feces (Jeschke et al., 2017), nitriles derived from benzylglucosinolate are further metabolized in *P.rapae*, leading to the release of cyanide and its subsequent detoxification by CAS (Stauber et al., 2012). In addition, numerous glucosinolate-specialists sequester and excrete glucosinolates (Müller et al., 2001; Aliabadi et al., 2002; Kazana et al., 2007; Yang et al., 2020). On the other hand, herbivores that are tolerant to glucosinolate defenses, like many generalist lepidopterans and leaf miner flies, detoxify glucosinolate-derived isothiocyanates by conjugating them with the tripeptide glutathione (γ-Glu–Cys–Gly) (Schramm et al., 2012; Gloss et al., 2014). However, this detoxification is nutritionally costly and can lead to a depletion of cysteine that negatively affects herbivore growth (Jeschke et al., 2016).

The two-spotted spider mite, *Tetranychus urticae* (Koch), is an extreme generalist herbivore that can feed on over one thousand plant hosts, including plants belonging to the Brassicaceae family (Migeon and Dorkeld, 2021). *Tetranychusurticae* is an example of a composite generalist herbivore whose individual populations perform well only on a subset of potential hosts (Fellous et al., 2014; Rioja et al., 2017). However, mites have an ability to adapt to originally unfavorable hosts in just 5–25 generations and overcome initially effective plant host defenses (Gould, 1979; Fry, 1989; Magalhaes et al., 2007; Wybouw et al., 2015; Salehipourshirazi et al., 2021). The expansion of gene families implicated in digestion, detoxification, and transport of xenobiotics and their dynamic expressional changes when mites shift to new plant hosts indicate that mites, like other generalist herbivores, can quickly reprogram their xenobiotic responses (Grbic et al., 2011; Herde and Howe, 2014; Schweizer et al., 2017; Salehipourshirazi et al., 2021). The detoxification potential of mites is enriched with genes that have been acquired through horizontal gene transfer (Wybouw et al., 2018). One such gene is *TuCAS* encoding an enzyme with cysteine synthase and β-cyanoalanine synthase activities. Its increased expression has been previously identified in mite populations that were adapted to *Phaseolus lunatus*, a plant host that accumulates cyanogenic glucosides (Wybouw et al., 2014). The ability of recombinant TuCAS to conjugate cyanide with cysteine, with high specificity, into β-cyanoalanine led to the hypothesis that increased expression of *TuCAS* enables *P. lunatus*-adapted mites to detoxify defensive cyanide (Wybouw et al., 2014).

**Figure 1:**
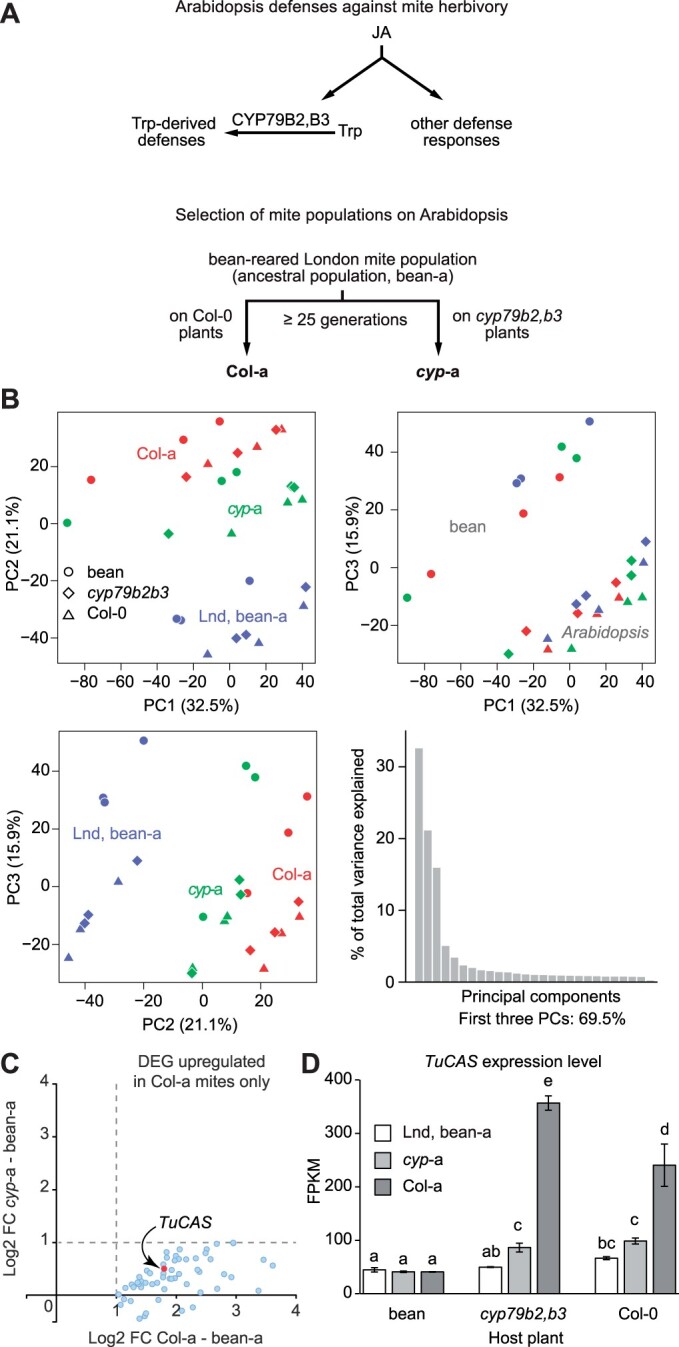
The high expression of β-cyanoalanine synthase (*TuCAS*) associates with mite adaptation to Arabidopsis. A, Simplified schematics of Arabidopsis induced defenses against mite feeding and selection of ancestral (Lnd, bean-a) mite population on Col-0 and *cyp79b2 cyp79b3* Arabidopsis plants, deriving Col-a and *cyp*-a populations respectively. B, A PCA of expression measures data for Col-a, *cyp*-a, and bean-a mite populations when moved to bean, Col-0, and *cyp79b2 cyp79b3 (cyp79b2,b3)* Arabidopsis plants. C, Differentially expressed genes upregulated in mites adapted to fully defended Col-0 plants but not in mites adapted to *cyp79b2 cyp79b3* Arabidopsis plants that lack CYP79B2 CYP79B3-derived defense metabolites. D, Expression levels of *TuCAS* in bean-a, Col-a, and *cyp*-a mites when fed on bean, *cyp79b2 cyp79b3* (*cyp79b2,b3*), and Col-0 Arabidopsis plants for 24 h. RNA-Seq experiment was performed in three biological replicates (*n* = 3). Data represent the mean of Fragments per Kilobase of transcript per Million mapped reads ± se. Different letters represent significant difference between means (Tukey’s HSD test, *P* < 0.05).

Arabidopsis is a nonhost plant to the reference London mite population (Grbic et al., 2011; Zhurov et al., 2014; Santamaría et al., 2017, 2019; Salehipourshirazi et al., 2021; Widemann et al., 2021). At least two classes of jasmonic acid (JA)-regulated defenses protect Arabidopsis plants against mite herbivory (Figure 1A; Salehipourshirazi et al., 2021; Widemann et al., 2021). Trp-derived indole glucosinolates are one class of defensive compounds (Widemann et al., 2021). They require myrosinase-catalyzed hydrolysis to form bioactive products with antifeedant effects against mites (Widemann et al., 2021). Besides indole glucosinolates, there is another class of JA-regulated defensive compound(s) that restrict mite herbivory on Arabidopsis. Their identity is currently not known. We recently demonstrated that a reference London bean-reared (*Phaseolus vulgaris*) mite population can adapt to Arabidopsis (Salehipourshirazi et al., 2021). In these experiments, London mites were selected on Columbia-0 (Col-0) fully defended wild-type (WT) plants, and *cyp79b2 cyp79b3* (*CYTOCHROME P450*, *FAMILY 79*, *SUBFAMILY B*, *POLYPEPTIDE 2* and *3*, *AT4G39950*, and *AT2G22330*) plants that are deficient in conversion of Trp to indole-3-acetaldoxime (IAOx), lack Trp-derived compounds but display the remaining JA-regulated defenses, resulting in Col-a and *cyp*-a mite populations, respectively (Figure 1A). Here, using these Arabidopsis-adapted mite populations, we initiated characterization of mite host-adaptive traits, focusing on those that enable mites to overcome indole glucosinolate-associated Arabidopsis defenses. Among genes associated with mite adaptation to indole glucosinolates we identified *TuCAS*. We show that *TuCAS* is required for mite adaptation to indole glucosinolates through its β-cyanoalanine synthase activity. Consistent with the role of β-cyanoalanine synthase in detoxification of HCN, we demonstrate that upon mite herbivory, Arabidopsis plants release HCN. Conversely, we identified the mite’s ability to mount cyanide detoxification counter defenses enabling mites to break Arabidopsis host resistance.

## Results

### The overexpression of *TuCAS* is associated with mite resistance to Arabidopsis defenses

Col-a and *cyp*-a mite populations were derived from an ancestral London strain (bean-a) that was selected on Col-0 and *cyp79b2 cyp79b3* Arabidopsis plants for ˃25 generations, respectively (Figure 1A). Both populations show similar fitness on *cyp79b2 cyp79b3* Arabidopsis plants lacking CYP79B2 CYP79B3-derived Arabidopsis defenses; however, Col-a mites significantly outperform *cyp*-a mites on Col-0 plants (Salehipourshirazi et al., 2021). We reasoned that exposure of Col-a mites to CYP79B2 CYP79B3-derived Arabidopsis defenses during the selection process resulted in development of the resistance traits that enable Col-a to overcome their toxicity. Conversely, these resistance traits are expected to be absent/less efficient in the ancestral bean-a and *cyp*-a populations that were not exposed to these defense compounds. Therefore, to identify genes associated with mite resistance to CYP79B2 CYP79B3-derived defenses, we performed a comparative transcriptomic analysis between Col-a, *cyp*-a, and bean-a mite populations. Prior to sample collection, Col-a and *cyp*-a mites that are maintained on Col-0 and *cyp79b2 cyp79b3* Arabidopsis plants, respectively, were transferred to bean plants for two generations to equalize the physiological effects of the rearing host on mite gene expression. Subsequently, Col-a, *cyp*-a, and bean-a mite populations were moved to bean, Col-0, and *cyp79b2 cyp79b3* Arabidopsis plants, and mite samples for RNASeq analysis were collected 24 h later.

Principal component analysis (PCA) of the complete data set consisting of 12,802 expressed genes indicated the robust effect of both mite strain and plant host experimental factors. The first three principal components explained 69.5% of the total data set variance (Figure 1B). The prominent effect of mite host-adaptation status as an experimental factor is seen in PC1 versus PC2 and PC2 versus PC3 plots. The separation of bean-a, *cyp*-a, and Col-a mite strains in these graphs correlates with the extent of host plant xenobiotic challenge to which these mite populations are adapted. The comparison PC1 versus PC3 clearly points to the effects of plant hosts on data set variance. As expected, Col-0 and *cyp79b2 cyp79b3* Arabidopsis plant hosts clustered together and away from bean as a factor, indicating that a small set of mite expressed genes is responsive to the presence of CYP79B2 CYP79B3-derived defenses. In the analysis of differential gene expression, we assessed the effects of mite adaptation status (ancestral bean-a, *cyp*-a, and Col-a) and host plant (bean, *cyp79b2 cyp79b3*, and Col-0) at absolute Log_2_ Fold Change (FC) of ≥1 and FDR-adjusted *P*-value ˂ 0.05. To identify genes associated with mite resistance to CYP79B2 CYP79B3-derived defenses we selected genes that are differentially expressed between Col-a and *cyp*-a mites independent of the host plant, and at the same time overexpressed in Col-a mites but not in *cyp*-a mites relative to the ancestral strain. This analysis identified a limited set of 59 genes (Figure 1C and Supplemental Table S1). One of them was *TuCAS*, a gene that encodes an enzyme with bifunctional cysteine synthase and β-cyanoalanine synthase activities (Wybouw et al., 2014). *TuCAS* was expressed at comparable basal levels in Col-a, *cyp*-a, and bean-a mites when they fed on bean plants for 24 h (Figure 1D). However, the expression of *TuCAS* increased when mites were transferred to Arabidopsis. Its expression in the ancestral bean-a mite population and *cyp*-a mites gradually increased when transferred to *cyp79b2 cyp79b3* and Col-0 leaves, with FC < 2 (Figure 1D). In contrast, *TuCAS* exhibited approximately nine- and six-fold upregulation in Col-a mites upon transfer from bean to *cyp79b2 cyp79b3* and Col-0 plants, respectively (Figure 1D). Therefore, *TuCAS* is expressed in response to Arabidopsis xenobiotics and has increased transcriptional plasticity in Col-a relative to *cyp*-a and bean-a mites.

### *TuCAS* is required for *T. urticae* adaptation to Arabidopsis

**Figure 2:**
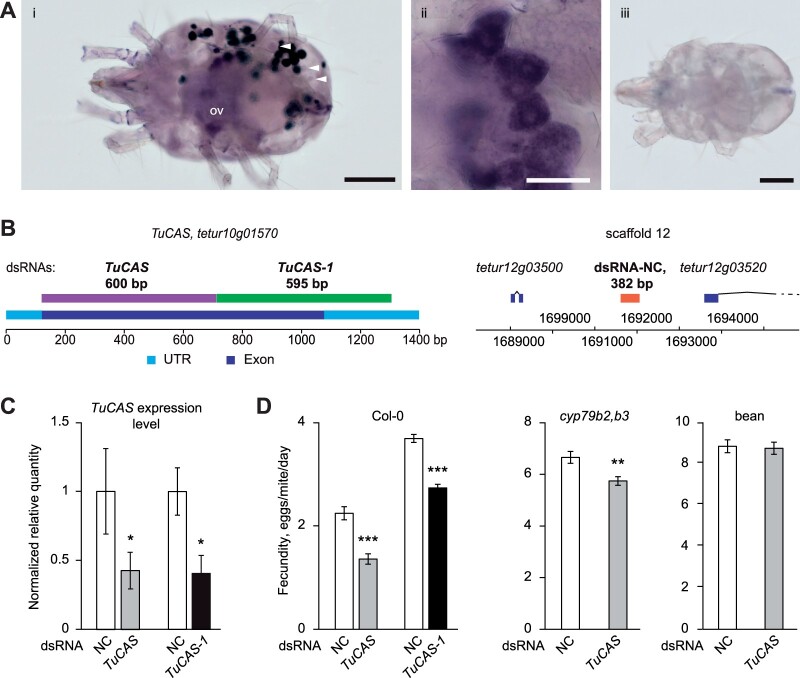
TuCAS is essential for *T. urticae* adaptation to Arabidopsis. A, The whole-mount in situ hybridization using the anti-sense (i, ii) and the sense (iii) probes of *TuCAS* in *T. urticae*; (i, iii) adult female, (ii) enlarged view of ovaries. Arrowheads in (i) point to digestive cells. ov, ovaries. B, Fragments used for the synthesis of dsRNAs. Schematics of the *TuCAS* locus with labeled DNA sequences used for the generation of dsRNA-*TuCAS* (600 bp) and dsRNA-*TuCAS-1* (595 bp), and the part of scaffold 12 of the *T*. *urticae* genome depicting the location of the 382 bp nontranscribed fragment that was used to synthesize dsRNA-NC. C, Relative level of *TuCAS* transcript normalized with *RP49* in *TuCAS* silenced Col-a mites (mean ± SE, *n* = 6, ANOVA *P < 0.05). D, The fecundity of dsRNA treated Col-a mites feeding on Col-0, *cyp79b2 cyp79b3* (*cyp79b2,b3*), and bean. Fecundity was measured over 2 d (3 and 4 postinoculation) and data are presented as the mean number of eggs laid by a female mite per day ± se (ANOVA **P < 0.01, ***P < 0.001). Experiments were performed in ten (for Col-0 and *cyp79b2 cyp79b3* plants) and five (for bean plants) biological replicates/trial and in three independent trials (*n* = 30, for Col-0 and *cyp79b2 cyp79b3* plants; and *n* = 15, for bean plants). Scale bar: A(i, iii) = 100 µm, A(ii) = 20 µm.

The high expression of *TuCAS* in Col-a mites upon transfer to *cyp79b2 cyp79b3* and Col-0 leaves may reflect a general increase in transcriptional plasticity of xenobiotically induced genes in Col-a mites or may point to the specific contribution of *TuCAS* to the resistance of Col-a mites to Arabidopsis defenses. To differentiate between these possibilities, we utilized a recently developed and optimized RNAi protocol (Suzuki et al., 2017a; Bensoussan et al., 2020) to silence *TuCAS* and determine if it is required for Col-a adaptation to Arabidopsis. *TuCAS* is a single gene in the mite genome encoded by the *tetur10g01570* locus (Grbic et al., 2011). It is constitutively expressed in adult spider mites (Figure 2A), including digestive cells that are considered to be the site for digestion and detoxification of dietary xenobiotics (Bensoussan et al., 2018). We synthesized two nonoverlapping dsRNA fragments, dsRNA-*TuCAS* (600 nt) and dsRNA-*TuCAS-1* (595 nt), that span a single exon and 3′-untranslated region of the *TuCAS* gene (Figure 2B). BLASTn search of the *T. Urticae* genome using *TuCAS* sequences as a query did not identify any sequences with a continuous identity with *TuCAS* longer than 19 nt. Therefore, *TuCAS* sequences are sufficiently dissimilar from other genes in the *T. urticae* genome and thus, *TuCAS* dsRNAs are not expected to have off-target effects. We also synthesized a dsRNA (382 nt) complementary to a nontranscribed genomic region that was used as a negative control (NC) (Figure 2B). Upon silencing, the expression of *TuCAS* was reduced by 56% (dsRNA-*TuCAS*) and 58% (dsRNA-*TuCAS-1*) in Col-a mites (Figure 2C). Reduced expression of *TuCAS* resulted in a significant reduction of fecundity in *TuCAS*-silenced Col-a mites fed on the Col-0 leaves (40% reduction in dsRNA-*TuCAS* and 26% in dsRNA-*TuCAS-1* treatments relative to dsRNA-NC treated Col-a mites) (Figure 2D). The similarity of RNAi effects obtained upon the application of two independent dsRNA-*TuCAS* fragments confirms the specificity of the requirement of TuCAS activity for the adaptation of Col-a mites to Col-0. When Col-a mites were treated with dsRNA-*TuCAS* and fed on *cyp79b2 cyp79b3* leaves, there was a modest but significant decrease in their fecundity (14% reduction), suggesting that Arabidopsis defenses counteracted by TuCAS are not exclusively dependent on the CYP79B2 CYP79B3-dependent pathway. Consistent with the requirement of TuCAS for mite adaptation to Arabidopsis, reduced *TuCAS* expression did not affect mite fitness when they fed on bean leaves (Figure 2D). Overall, our data indicate that *TuCAS* is required for mite adaptation to Arabidopsis, enabling mites to primarily counteract CYP79B2 CYP79B3-dependent and to a smaller extent, CYP79B2 CYP79B3-independent Arabidopsis defenses.

### Cysteine synthase activity of TuCAS is not required for mite adaptation to Arabidopsis

**Figure 3:**
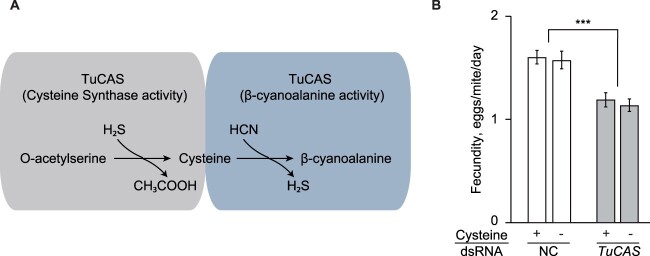
Cysteine synthase activity of TuCAS in Col-a mites is not associated with mite adaptation to Arabidopsis. A, Schematic representation of TuCAS dual enzymatic functions. B, The fecundity (recorded over 2 d [3 and 4 postinoculation]) of Col-a mites treated with dsRNA-NC (NC) or dsRNA-*TuCAS* (*TuCAS*) feeding on Col-0 leaves supplemented with cysteine (+) or water (−). Data represent the mean number of eggs laid by a female mite per day ± se. The experiment was performed in five biological replicates/trial and in three independent trials, *n* = 15. Significance of dsRNA treatment detected through ANOVA, ***P < 0.001.

TuCAS is a bifunctional enzyme that has cysteine synthase activity (enabling cysteine biosynthesis from H_2_S and *O*-acetylserine) and β-cyanoalanine synthase activity (required for the synthesis of β-cyanoalanine from cyanide (HCN) and cysteine) (Figure 3A). Of the two enzymatic activities, β-cyanoalanine synthase activity is strongly favored (Wybouw et al., 2014; Daneshian et al., 2022). Cysteine is one of the structural amino acids of glutathione that is essential for glutathione-*S*-transferase (GST)-dependent detoxification of xenobiotic compounds. Col-0 WT plants have a greater complement of defenses relative to *cyp79b2 cyp79b3* plants. Thus, it is conceivable that Col-a mites gained an ability to synthesize greater amounts of cysteine as an adaptation to a greater requirement of the glutathione conjugation of Arabidopsis xenobiotic compounds. To test the possibility that TuCAS protects Col-a mites from cysteine depletion, we used petiole-infiltration to administer either 1 mM cysteine solution or water as a control into Col-0 leaves. To ensure that supplemented leaves had elevated levels of cysteine relative to water-treated leaves, whole leaf extracts were subject to LCMS analysis. While the level of cysteine in untreated leaves was below the level of detection of our instrumentation, leaves with administered cysteine had 117 ± 73 pmol·mg^−1^ FW cysteine equivalents (measured as cystine; see “Materials and methods”), which is approximately seven-fold greater than reported for WT Arabidopsis leaves (Krueger et al., 2009). Treated leaves were subsequently infested with Col-a mites that had been treated with dsRNAs complementary to *TuCAS* or the NC genomic region. If the cysteine synthase activity of TuCAS contributes to the resistance of Col-a mites against Arabidopsis defenses, its requirement is expected to be relieved by the externally provided cysteine. In line with the results shown in Figure 2D, silencing of *TuCAS* reduced the fecundity of Col-a mites exposed to Col-0 defenses (Figure 3B). However, mite fecundity was comparable when dsRNA-treated Col-a mites fed on leaves supplemented with cysteine or water (Figure 3B), suggesting that the cysteine synthase activity of TuCAS is not required for mite adaptation to Arabidopsis.

### Col-a mites have greater tolerance to cyanide

**Figure 4:**
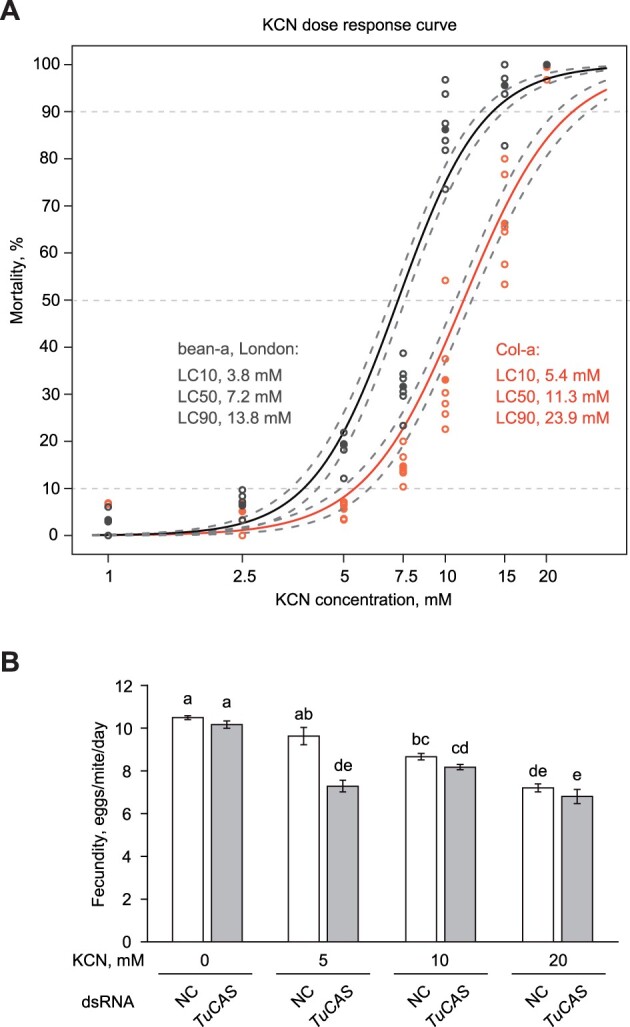
β-cyanoalanine synthase activity of TuCAS in Col-a mites is associated with their adaptation to Arabidopsis. A, KCN dose-response curves with 95% confidence intervals for bean-a (London) and Col-a mites. Mite mortality was scored 24 h after KCN treatment. The experiment was performed in two biological replicates and three independent trials, *n* = 6. Filled dots represent an average of six samples. B, The fecundity (recorded over 2 d [3 and 4 postinoculation]) of Col-a mites treated with dsRNA-NC (NC) or dsRNA-*TuCAS* (*TuCAS*) feeding on bean leaf discs coated with water or 5, 10, or 20 mM KCN. Data represent the mean number of eggs laid by a female mite per day ± se. The experiment was performed in three biological replicates and three independent trials, *n* = 9. Significant interaction between KCN treatment and dsRNA treatment was detected by ANOVA, *P* < 0.001. Different letters represent significant difference between means (Tukey’s HSD test, *P* < 0.05).

To test if the high expression of *TuCAS* in Col-a mites confers their tolerance to HCN we compared the susceptibility of the ancestral bean-a and Col-a mite populations to cyanide. Using direct delivery of KCN to mites (Ghazy et al., 2020), we determined the susceptibility of bean-a and Col-a mites to a range of KCN concentrations. Both bean-a and Col-a mites were insensitive to 1 and 2.5 mM KCN (Figure 4A). Higher KCN concentrations caused a dose-dependent increase in mite mortality that reached 100% at ≥20 mM in both bean-a and Col-a mite populations (Figure 4A). However, the dose-dependent mortality was shifted toward higher KCN concentrations in Col-a relative to bean-a mites, indicating that Col-a mites have greater tolerance to cyanide (Figure 4A). If increased cyanide resistance in Col-a mites requires a high level of *TuCAS* expression, then silencing of *TuCAS* should restore their cyanide sensitivity. To test this hypothesis, we used the leaf coating method of HCN delivery, previously shown to enable compound delivery to mites through ingestion (Suzuki et al., 2017b). As leaf coating is a less efficient way of compound delivery relative to direct mite exposure used in Figure 4A, we first identified the highest asymptomatic KCN concentration using mite fecundity as a fitness parameter. As seen in Figure 4B, the fecundity of Col-a mites was significantly reduced on bean leaf discs coated with 10 and 20 mM KCN. However, the application of 5 mM KCN did not affect mite fecundity. Both dsRNA treatment and KCN application had a highly significant effect on Col-a mite fecundity (ANOVA, *P* ≪ 0.001). Consistent with data shown in Figure 2D, silencing of *TuCAS* had no effect on fecundity on water-treated bean leaf disks. While feeding on 5 mM KCN treated disks was asymptomatic (no decrease in NC-mite fecundity), silencing of *TuCAS* at this KCN concentration had a dramatic 25% reduction in fecundity, suggesting that TuCAS β-cyanoalanine synthase activity is required for cyanide tolerance of Col-a mites. Increasing concentrations of KCN resulted in a linear decrease in mite fecundity. At these higher KCN concentrations the contribution of *TuCAS* knockdown on mite fecundity was not significant.

### TuCAS protects Col-a mites against indole glucosinolate-dependent cyanide

**Figure 5:**
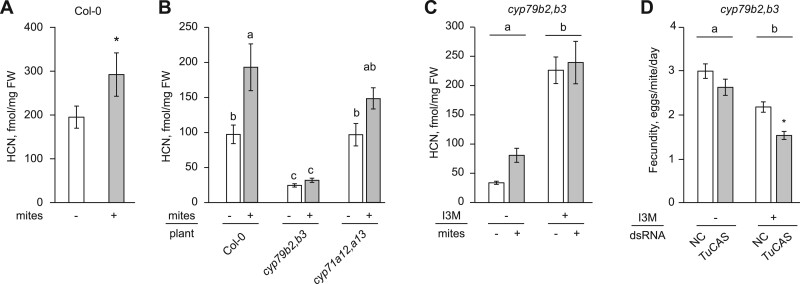
Cyanide synthesis in Arabidopsis upon mite feeding requires CYP79B2 CYP79B3-derived indole glucosinolates. A and B, Cyanide levels in Col-0, *cyp79b2 cyp79b3* (*cyp79b2,b3*), and *cyp71a12 cyp71a13* (*cyp71a12,a13*) plants with and without bean-a mite feeding. Data represent the mean ± SE. The experiment was performed in five independent trials (1 sample/treatment/trial), *n* = 5. In (A), the significant effect of mite feeding detected by ANOVA is represented as an asterisk. In (B), a significant interaction between plant genotype and mite feeding was detected by ANOVA and was followed by a Tukey’s HSD test with letters representing significant differences between means, *P* < 0.05. C, Cyanide levels in *cyp79b2 cyp79b3* (*cyp79b2,b3*) plants supplemented with 2.4 mM I3M for 24 h with and without bean-a mite infestation. Data represent the mean ± SE. The experiment was performed in six independent trials (one sample/treatment/trial), *n* = 6. Significant effects of I3M supplementation detected by ANOVA are represented by different letters at *α* = 0.05. D, The fecundity (recorded on Day 3 postinoculation) of Col-a mites feeding on *cyp79b2 cyp79b3* (*cyp79b2,b3*) leaves supplemented with 2.4 mM I3M (for 24 h). Col-a mites were treated with either dsRNA-NC or dsRNA-*TuCAS.* Data represent the mean number of eggs laid by a female mite per day ± se. The experiments were performed in five biological replicates/trial and in three independent trials, *n* = 15. No interaction between I3M and dsRNA treatment was detected by ANOVA, but main effects of I3M supplementation (represented by different letters) and dsRNA were significant at *α* = 0.05. Differences between dsRNA treatments within I3M supplementation regimes were detected by unpaired Student’s *t* tests (*P < 0.05).

**Figure 6:**
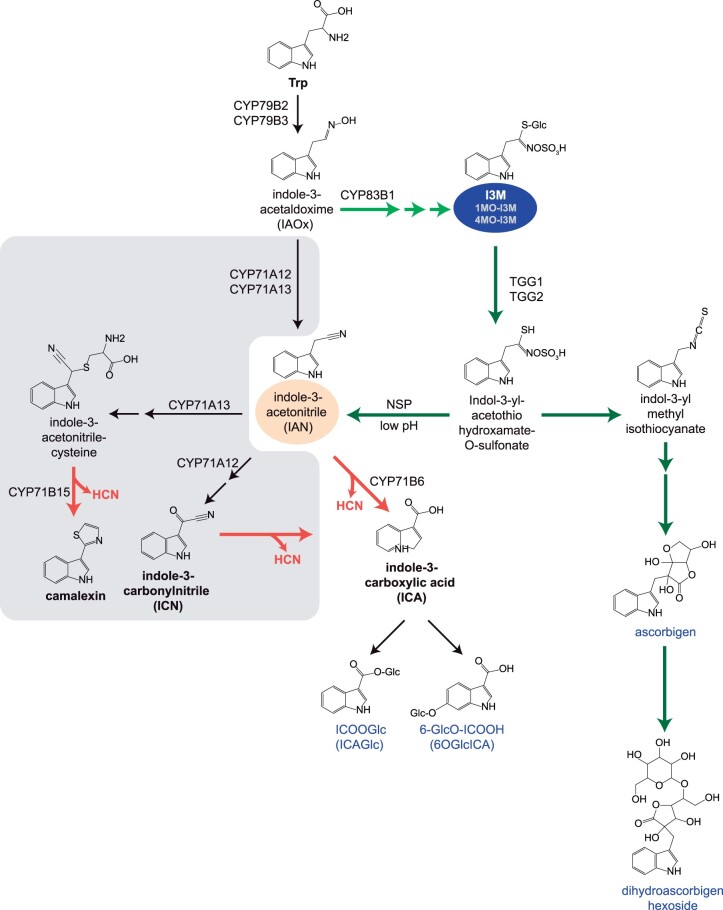
Model of the biosynthetic network of indolic metabolites in Arabidopsis. Indole glucosinolates, I3M, and its derivatives 1-methoxy-I3M and 4-methoxy-I3M (1MO-I3M and 4MO-I3M) are circled in blue. Indole glucosinolate biosynthetic and breakdown pathways are marked by light and dark green arrows, respectively. Metabolites that were putatively identified as indole glucosinolate breakdown products in Arabidopsis leaves upon mite feeding (Widemann et al., 2021) are marked in blue. Proposed cyanogenic pathways, dependent on CYP71A12 and/or CYP71A13 leading to the synthesis of camalexin and ICN are colored in gray. Reactions expected to lead to cyanide release are marked as orange arrows (Böttcher et al., 2009; Böttcher et al., 2014; Rajniak et al., 2015). Multiple arrows indicate multiple enzymatic reaction steps. NSP, nitrile specifier protein; TGG1/2, THIOGLUCOSIDE GLUCOHYDROLASE 1 and 2 myrosinases.

If β-cyanoalanine synthase activity of TuCAS is required for mite adaptation to CYP79B2 CYP79B3-dependent Arabidopsis defenses, then mites should be exposed to cyanide upon their interaction with Arabidopsis. However, the existence and the potential source of cyanide that may protect Arabidopsis against mite herbivory are not known. We first tested if cyanide levels in Col-0 leaves change in response to mite feeding. As seen in Figure 5A, untreated Col-0 leaves accumulate basal levels of cyanide that significantly increase upon infestation by bean-a mites. Furthermore, an ∼80% reduction of cyanide basal levels and lack of the additional cyanide accumulation upon bean-a mite feeding on *cyp79b2 cyp79b3* relative to Col-0 leaves (Figure 5B), indicate that cyanide release requires CYP79B2 CYP79B3-dependent pathway(s). Camalexin, indole-3-carbonylnitrile (ICN) and indole-3-carboxylic acid (ICA) are CYP79B2 CYP79B3-dependent pathways that were previously implicated in cyanide formation (Figure 6; Böttcher et al., 2009, 2014; Rajniak et al., 2015). However, the comparable cyanide levels in Col-0 and *cyp71a12 cyp71a13* double mutant plants (Figures 5B and 6) indicate that formation of indole-3-acetonitrile (IAN) via CYP71A12 and CYP71A13 and downstream camalexin and ICN pathways are not required for cyanide syntheses in response to mite feeding. To test if the indole glucosinolate-dependent pathway contributes to cyanide synthesis, we complemented *cyp79b2 cyp79b3* mutant leaves with 2.4 mM 3-indolylmethyl glucosinolate (I3M) solution, previously shown to reconstitute CYP79B2 CYP79B3-derived defenses (Widemann et al., 2021). As seen in Figure 5C, the addition of I3M resulted in increased cyanide levels in *cyp79b2 cyp79b3* mutant leaves regardless of mite challenge, demonstrating that indole glucosinolates are sufficient for cyanide formation. If cyanide is a constituent of I3M-dependent Arabidopsis defenses against mites, then TuCAS should enable Col-a mites to counteract cyanide poisoning in *cyp79b2 cyp79b3* mutant leaves complemented with I3M. In line with a low accumulation of cyanide in *cyp79b2 cyp79b3* plants, silencing of *TuCAS* resulted in only a small nonsignificant decrease in Col-a mite fecundity in the absence of I3M (Figure 5D). However, upon I3M supplementation of *cyp79b2 cyp79b3* leaves, silencing of *TuCAS* led to a significant decrease of Col-a mite fitness (Figure 5D) demonstrating that TuCAS protects Col-a mites against I3M-dependent cyanide defenses.

## Discussion

Glucosinolates are considered to be the main toxic compounds in Arabidopsis that restrict herbivory of a wide range of arthropods including the two-spotted spider mite, *T. urticae* (Wittstock and Halkier, 2002; Wittstock et al., 2003; Zhurov et al., 2014; Widemann et al., 2021). Using differential sensitivity of Col-a relative to *cyp*-a and bean-a mites to CYP79B2 CYP79B3-dependent defenses (Figure 1; Salehipourshirazi et al., 2021), we identified *TuCAS—*a gene that has been independently acquired by mites and lepidopteran insects from bacteria via horizontal gene transfers (Wybouw et al., 2014; van Ohlen et al., 2016; Herfurth et al., 2017; Li et al., 2021). In both classes of herbivores, *CAS* expression is elevated when Arabidopsis glucosinolates are encountered. In lepidopteran generalists (e.g. the tobacco budworm *Heliothis virescens*), like in bean-a and *cyp*-a mites, *CAS* expression undergoes a modest increase in response to Arabidopsis glucosinolates (Schweizer et al., 2017). However, in a specialist (e.g. the large white *Pieris brassicae*) and in Col-a mites, the exposure to glucosinolates leads to high *CAS* expression (Figure 1; Schweizer et al., 2017), indicating that Col-a mites behave like Brassicaceae-specialized herbivores in regard to *TuCAS* responsiveness to glucosinolates.

TuCAS is a bifunctional enzyme with cysteine synthase and β-cyanoalanine synthase activities, albeit carried out with different efficiency (Figure 3A; Wybouw et al., 2014; Daneshian et al., 2022). We tested if the cysteine synthase activity of CAS may be required to offset cysteine depletion that may result from detoxification of xenobiotic compounds through glutathione conjugation (Schramm et al., 2012; Gloss et al., 2014; Jeschke et al., 2016). However, elevated cysteine levels did not eliminate the requirement of TuCAS activity when Col-a mites were exposed to Col-0 defenses (Figure 3), suggesting that the cysteine synthase activity of TuCAS is dispensable for mite adaptation to Arabidopsis. This is consistent with the low cysteine synthase activity of TuCAS (Wybouw et al., 2014; Daneshian et al., 2022). Instead, several lines of evidence suggest that the β-cyanoalanine synthase activity of TuCAS that catalyzes the reaction between HCN and cysteine to form β-cyanoalanine may be required for mite adaptation to indole glucosinolates. First, Col-a mites have greater tolerance to exogenously supplied cyanide than bean-a mites (Figure 4). Second, cyanide was detected in Arabidopsis leaves and its levels increased upon mite feeding (Figure 5A). Third, indole glucosinolates are sufficient for the release of cyanide in Arabidopsis leaves (Figure 5C). Fourth, TuCAS is required for high mite performance in the presence of the indole glucosinolates (Figure 5D). Cumulatively, our data suggest that indole glucosinolate-dependent cyanide protects Arabidopsis plants against mite herbivory and that in response, Col-a mites utilize the β-cyanoalanine synthase activity of TuCAS to counter cyanide toxicity.

CYP79B2 CYP79B3 convert Trp into IAOx that is further processed by CYP71A12, CYP71A13 and CYP83B1 to initiate the biosynthesis of camalexin, ICN, ICA, and indole glucosinolates, respectively (Figure 6; Halkier and Gershenzon, 2006; Rauhut and Glawischnig, 2009; Rajniak et al., 2015; Müller et al., 2019; Pastorczyk et al., 2020). CYP71A12 and CYP71A13 direct IAOx toward the synthesis of IAN that is then used as a substrate by CYP71A12, CYP71A13, and CYP71B6 to derive camalexin, ICN, and ICA, respectively, through pathways predicted to lead to cyanide release (Figure 6). For example, the final conversion of the IAN-cysteine conjugate to camalexin by CYP71B15 is expected to release equimolar amounts of cyanide (Böttcher et al., 2009). Likewise, the spontaneous hydrolysis of ICN and 4-OH-ICN yielding ICA and 4-OH-ICA is predicted to produce HCN (Rajniak et al., 2015). In addition, cyanide release, at least in vitro, occurs upon the conversion of IAN to its oxidized derivatives indole-3-carbaldehyde and ICOOH/ICA in reactions carried out by CYP71B6 (Böttcher et al., 2014; Müller et al., 2015, 2019). However, CYP71A12 and CYP71A13 are dispensable for Arabidopsis defenses against mite herbivory (Widemann et al., 2021) and for the synthesis of mite-induced cyanide (Figure 5B). Instead, the reconstitution of cyanide release in *cyp79b2 cyp79b3* leaves by I3M supplementation indicates that mite-induced cyanide is indole glucosinolate-dependent (Figure 5B). If indole glucosinolate-dependent cyanide is formed by any of the above-mentioned pathways, then the breakdown of indole glucosinolates should lead to the production of IAN at the expense of indol-3-yl-methyl isothiocyanate. In that case, Arabidopsis nitrile specifier proteins (NSPs; Burow et al., 2009; Wittstock and Burow, 2010) and/or acidification of the cytosol potentially resulting from the formation of reactive oxygen species upon mite feeding (Santamaría et al., 2018) will enable the formation of IAN from indole glucosinolates (Latxague et al., 1991). Consistent with this possibility, the analysis of indole glucosinolate breakdown products in Col-0 leaves upon mite feeding (Widemann et al., 2021) identified features that may correspond to ICOOGlc and 6-GlcO-ICOOH (Widemann et al., 2021), raising the possibility that indole glucosinolate breakdown in mite-infested Col-0 leaves could lead to the formation of IAN and the synthesis of ICA, with the concomitant release of cyanide. Cyanide release in planta is also expected as a by-product of ethylene biosynthesis (Peiser et al., 1984; Yip and Yang, 1988). Ethylene-dependent cyanide release is expected in both Col-0 and *cyp79b2 cyp79b3* leaves and could account for the CYP79B2 CYP79B3-independent requirement of *TuCAS* in Col-a mites (Figure 2). Given that TuCAS has broader enzymatic activities beyond utilization of cyanide as a substrate (Daneshian et al., 2022), we cannot exclude the possibility that some other plant compounds (e.g. upstream Trp-derived metabolites that may accumulate in *cyp79b2 cyp79b3* leaves) are detoxified by TuCAS.

Alternatively, the glucosinolate-dependent cyanide could be generated in the mite gut, similarly to cyanide formation reported for Brassicaceae specialists belonging to the Pieridae family, for example, *P.rapae* and *P. brassicacae* (Stauber et al., 2012). These herbivores express the NSP, which when combined with plant myrosinases redirects glucosinolate hydrolysis toward nitrile instead of isothiocyanate formation (Wittstock et al., 2004). Nitriles derived from the benzylglucosinolate undergo further metabolism in the caterpillars’ gut, during which they release cyanide that is subsequently detoxified by CAS (Stauber et al., 2012). Mite feeding induces de novo synthesis of indole glucosinolates in Col-0 leaves and triggers their in planta myrosinase breakdown (Widemann et al., 2021). Plant or mite-generated nitriles may undergo further metabolism in the mite gut to derive cyanide. In that case, TuCAS protects mites from self-generated cyanide produced from the indole glucosinolate-derived nitriles.

Besides HCN, other indole glucosinolate breakdown products are known to have defensive effects against herbivores (Kim et al., 2008). The supplementation of *cyp79b2 cyp79b3* leaves with indole glucosinolates generates HCN (Figure 5C), as well as 192 other potential breakdown products and derivatives whose function in Arabidopsis defense against mites remains to be demonstrated (Widemann et al., 2021). We hypothesize that multiple indole glucosinolate breakdown products and derivatives may contribute to Arabidopsis defense against mites. In addition, we have identified a prominent role of CYP79B2 CYP79B3-independent defenses that protect Arabidopsis against mite herbivory (Widemann et al., 2021). Consequently, our expectation is that TuCAS is not the only enzyme ensuring mite adaptation to Arabidopsis. We have previously identified the requirement of both GST and in particular cytochrome P450 monooxygenase enzymatic activities for mite adaptation to Arabidopsis (Salehipourshirazi et al., 2021). Thus, the genetic complexity of mite adaptation to Arabidopsis is expected to mirror the complexity of Arabidopsis defenses that mites must overcome in order to successfully establish themselves on this plant host. While TuCAS is encoded by a single gene in the *T. urticae* genome, other resistance genes (e.g. GSTs and CYPs) reside within gene families that have expanded in the *T. urticae* genome, increasing the difficulty of their identification using an RNAi approach.

In conclusion, our data indicate that upon feeding on Arabidopsis plants mites are exposed to indole glucosinolate-dependent cyanide. In response, Arabidopsis-adapted mites utilize the β-cyanoalanine synthase activity of TuCAS to counteract cyanide toxicity. Our work highlights mite’s ability to respond to cyanide toxicity by activating resistant traits that enable this extreme polyphagous herbivore to exploit cyanogenic host plants.

## Materials and methods

### Plant materials and growth conditions

Arabidopsis (*A.thaliana*) Col-0 WT seeds were obtained from the Arabidopsis Biological Resource Center (Ohio State University). The seeds of *cyp79b2 cyp79b3* (Zhao et al., 2002) and *cyp71a12 cyp71a13* (Müller et al., 2015) mutants were kindly provided by B. A. Halkier (University of Copenhagen, Denmark) and E. Glawischnig (Technical University of Munich, Germany), respectively. All plants were grown under controlled growth conditions at 24°C with 50% relative humidity and a short-day photoperiod (10-h:14-h [light:dark]) in 100–150 μmol m^−2^ sec^−1^ white fluorescent light. The seeds of an acyanogenic cultivar California Red Kidney beans (*P.vulgaris*) were purchased from Stokes, Thorold, Ontario, and were grown at 25°C with 55% relative humidity and a 16-h/8-h (light/dark) photoperiod.

### Spider mite strains and rearing conditions

The London ancestral mite population (bean-a) was maintained on bean plants at 25°C, 55% relative humidity, and a 16-h:8-h (light:dark) photoperiod for more than 10 years (Grbic et al., 2011). Col-a and *cyp*-a mite populations, adapted to Col-0 and *cyp79b2 cyp79b3* Arabidopsis plants, respectively (Salehipourshirazi et al., 2021), were maintained under controlled conditions at 24°C, 50% relative humidity and a 10-h/14-h (light/dark) photoperiod. Prior to every experiment, Arabidopsis-adapted mites were reared on bean plants alongside with London mite population for two generations, to eliminate the environmental and plant–host physiological effects.

### Transcriptome analysis by RNA-Seq

Bean-a, *cyp*-a, and Col-a spider mite females were transferred from their respective plant hosts to detached bean leaves where they were maintained for 2 weeks. Subsequently, adult female mites from bean-reared populations were used to inoculate 1-week-old bean, and 4- and 5-week old *cyp79b2 cyp79b3* and Col-0 plants. After 24 h, three samples of 100 spider mite females were collected for each treatment, frozen in liquid nitrogen, and stored at −80°C until RNA extraction. Total RNA was extracted using RNeasy Mini Kit (Qiagen, Venlo, Limburg, Netherlands), including on-column DNase treatment, following the manufacturer’s instructions. The quality and quantity of the extracted RNA were determined using a Nanodrop ND-2000c spectrophotometer (Thermo Scientific, Waltham, MA, USA). Strand specific paired-end (2 × 150 bp) sequencing was conducted according to Illumina TruSeq protocol (Illumina, San Diego, CA, USA). The transcriptome sequencing of all 27 libraries was performed on a single sequencing lane on an Illumina HiSeq2500 Genome Analyzer (Illumina, San Diego, CA, USA) platform yielding 8–17 million mapped fragments per library. Reads were mapped to the reference *T. urticae* genome (assembly 2009-09-29; Grbic et al., 2011) using STAR aligner (Dobin et al., 2013) version 2.5.2b in a single-pass mode with annotation allowing only unique mapping, up to five mismatches per read mapped, a minimum intron size of 20 bp, a maximum intron size of 15,000 bp, and outFilterMatchNminOverLread of 0.5. Read counts were generated at the level of gene locus using HTSeq version 0.6.0 in “union” mode (Anders et al., 2015) against the *T. urticae* genome annotation version 2016-06. Genes expressed at the level at or above 1 fragment count per million (CPM) reads in at least three samples were considered for the subsequent analysis. Analysis of differential gene expression was performed using voom/limma workflow for genes that demonstrated expression levels of at least 1 CPM in at least three samples (Law et al., 2014). Additional analysis and figures were performed and generated using R (R Core Team, 2013) and BioConductor (Gentleman et al., 2004).

### dsRNA preparation and application

Two nonoverlapping dsRNAs (*TuCAS*, 600 nt) and (*Tu-CAS-1*, 595 nt) are complementary to the transcribed sequence of *TuCAS* (*tetur10g01570*). The NC dsRNA (referred to as NC) is complementary to the nontranscribed intergenic region (1690614.1690995) of genomic scaffold 12 (Grbic et al., 2011; Sterck et al., 2012). A BLAST search against the *T. urticae* genome confirmed that dsRNA sequences were unique (Altschul et al., 1997). dsRNAs were synthesized according to the protocol described in (Suzuki et al., 2017a). dsRNAs were delivered to mites following the protocol described previously (Bensoussan et al., 2020). Briefly, 50 newly molted mites (Suzuki et al., 2017b) were soaked in 50 μL of dsRNA solution (500 ng/µL) supplemented with 0.1% (v/v) tween-20 and 6% (v/v) food-grade blue dye erioglaucine (McCormick, Sparks Glencoe, MD, USA) at 20°C for 24 h. Subsequently, mites were washed twice with double distilled water and allowed to recover on bean leaves. Mites that had blue color in the posterior midgut were selected and used for all experiments.

### RT-qPCR analysis

Total RNA was extracted from a pooled sample of 40 mites using RNeasy Mini Kit (Qiagen). Extracted RNA (1 µg) was reverse transcribed into cDNA using Maxima First Strand cDNA Synthesis Kit (Thermo Fisher Scientific). Reverse transcription quantitative PCR (RT-qPCR) was performed in three individual technical replicates for each RNA sample with Maxima SYBR green ROX qPCR master mix using primers listed in Supplemental Table S2. The expression of *ribosomal protein 49* (*RP49*, *tetur18g03590*), shown to be uniform under our experimental conditions (Bensoussan et al., 2020), was used to derive the relative expression of *TuCAS* in dsRNA-NC and dsRNA-*TuCAS* treated mites. Normalized relative quantity (NRQ) was calculated by using NRQ = (1 + ER)^CtR^/(1 + ET)^CtT^ where ER and ET are the primer efficiency of *RP49* and *TuCAS*, respectively. One biological replication per treatment was collected per experimental trial in six trials. For statistical analysis, NRQ values were Log2 transformed and analyzed by a two-way ANOVA using the variables dsRNA treatment, and experimental trial as a blocking factor (without interaction) (Brady et al., 2015). Significance level for all statistical analyses for all experiments was set at 0.05.

### In situ hybridization

Whole-mount in situ hybridization was performed with DIG-labeled probes (Dearden et al., 2002). A Zeiss AxioCam HRc 412-312 camera mounted on a Zeiss Axioplan II microscope was used to capture images.

### Mite fecundity assay

Mite fecundity assay on Arabidopsis leaves was performed as described in Widemann et al. (2021). Briefly, the petiole of fully expanded leaf from 5-week-old Arabidopsis plants was cut and submerged in 10 mL of water contained in a small Petri plate covered with parafilm. Each leaf was inoculated with 10 mites and the Petri plate was closed with a vented lid to ensure mite containment. Petri plates were kept under standard mite rearing conditions and leaves were replaced every other day. For fecundity assays on bean plants, leaf discs (15 mm) were cut using a hole puncher and placed on water-soaked cotton inside the polystyrene cups (V-9, As-one, Osaka, Japan) covered with ventilated lids. Each disc was inoculated with ten mites and was kept under standard mite rearing conditions. Discs were replaced every other day. The fecundity data represent the mean number of eggs deposited per mite over 2 d, except for data shown in Figure 5D where eggs deposited per mite were determined over 3 d. Fecundity assays were performed in 10 biological replicates/trial when mites fed on Arabidopsis leaves and in 5 biological replicates/trial when on bean leaf disks. Experiments were replicated in at least three independent experimental trials. Statistical analysis was performed using a two-way ANOVA with experimental trial and dsRNA treatments as main factors (without interaction) (Brady et al., 2015).

### KCN toxicity bioassays

Square-cut pieces (49 mm^2^) of Kimwipe placed inside the small Petri plate were saturated with 10 µL of aqueous solutions containing different concentrations of KCN (1, 2.5, 5, 7.5, 10, 15, and 20 mM). Around 40 3-d-old adult female mites were placed onto each Kimwipe and were gently oriented with the dorsal side up using a fine brush. Another piece of square-cut Kimwipe was used to cover mites and an additional 10 µL of the solution was applied, completely soaking the Kimwipes. Petri plates were covered with a lid, sealed with parafilm, and incubated for 20 h at 20°C. Postincubation, Kimwipes were transferred to detached bean leaves placed on water-soaked cotton. Mite mortality was scored 4 h later. Mites were considered alive if they could walk normally after they were prodded with a fine brush. A total of six replicates (two biological replicates per trial and in three independent trials) of six KCN concentrations and water control were tested. The KCN dose–response curve was constructed using R package drc (Ritz et al., 2016). For fecundity assays, bean leaf discs (15 mm) were painted with KCN solutions (Suzuki et al., 2017b). Twenty one microliters of 5, 10, and 20 mM KCN solutions with a water control were placed on water-soaked cotton inside a polystyrene cup (V-9, As-one, Osaka, Japan) with a ventilated lid, and were infested with ten mites each. Col-a mites treated with dsRNA-*TuCAS* and dsRNA-NC were allowed to recover on a bean leaf for 24 h before being transferred to a bean leaf disk coated with KCN. Leaf discs were kept under standard mite rearing conditions and were replaced every second day. The fecundity data represent the mean number of eggs deposited per mite on the third and fourth days of the experiment. The experiment was performed in three biological replicates per trial and in three independent trials. Statistical analysis was performed using a three-way ANOVA with experimental trial (blocking factor), KCN treatment, and dsRNA treatment as main factors, including an interaction term between dsRNA treatment and KCN treatment (Brady et al., 2015) followed by Tukey’s honestly significant difference (HSD) test upon detection of a significant interaction.

### Cysteine and I3M supplementation assays

Fully developed leaves of 5-week-old Col-0 and *cyp79b2 cyp79b3* plants were excised and their petioles were submerged in aqueous solutions of 1 mM cysteine (catalog number 168149; Sigma Aldrich) for 6 h and 2.4 mM I3M (I3M-glucobrassicin, catalog number 2525; Extrasynthese, France) for 24 h, respectively. Thereafter, leaves were inserted in a small Petri plate covered with parafilm and containing 10 mL of water and were inoculated with 10 mites. The number of eggs laid by ten mites was determined after 2 d (third and fourth day of the experiment) and 3 days (second, third, and fourth day of the experiment) for cysteine and I3M supplementation, respectively. Data are presented as number of eggs deposited per mite per day. The experiment involving cysteine supplementation was performed in three experimental trials (four biological replications/trial) and analyzed by three-way ANOVA with experimental trial (blocking factor), supplementation (± cysteine), and dsRNA treatment used as main effects including an interaction term between cysteine and dsRNA treatments. The experiment involving I3M supplementation for cyanide quantification was performed in six experimental trials (one biological replication per I3M treatment and mite treatment combination per trial) and was analyzed by three-way ANOVA using trial (blocking factor), I3M treatment and mite treatment as main factors including an interaction between I3M and mite treatments. The fecundity assay of I3M supplemented leaves with dsRNA treated mites was performed in five biological replicates/trial and three independent trials. Data were then analyzed by three-way ANOVA using trial (blocking factor), I3M supplementation, and dsRNA treatment as main effects including an interaction between I3M supplementation and dsRNA treatment term.

### Cysteine extraction and quantification

Verification of cysteine uptake by petiole infiltration was demonstrated by supplementing fully developed leaves of 5-week-old Col-0 with 1 mM cysteine (catalog number 168149; Sigma Aldrich) or water. Following 6 h of supplementation, the leaves were transferred to small Petri plates covered with parafilm containing 10 mL of water for 24 h before sample collection and weighing. Amino acids were extracted from frozen tissues in 0.1 M HCl containing l-Trp-(*indole*-d_5_) (25 µg mL^−1^; Sigma-Aldrich, 615862) as an internal standard, with a leaf fresh weight (F.W.)/buffer volume ratio of 100 mg mL^−1^. After manually grinding the biological sample in the buffer with a pestle, metabolites were further extracted by vortexing (1 min) and then by sonication (10 min). Debris was removed by two successive centrifugations at 16,160 × *g*, and the supernatant was analyzed by high-performance liquid chromatography on an Agilent 1260 LC system (Agilent Technologies, Santa Clara, CA, USA) equipped with a ZIC-HILIC column (2.1 × 100 mm, 3.5 μm; Merck, Kenilworth, NJ, USA). An aliquot (20 μL) of the prepared sample was separated at 400 µL min^−1^ and 30°C by applying the following gradient: 0 to 5 min, 100% B (90% acetonitrile v/v, 0.1% v/v formic acid in Milli-Q H_2_O); 13 min, 55% B, 45% A (5 mM NH_4_Ac, pH 4); hold at 55% B for 2 min; 15.5 min, 100% A; hold at 100% A for 2.5 min; 19 min 100% B. An 11-min postrun equilibration was completed at 100% B between each sample. ESI-TOF parameters: drying gas at 325°C, 12 mL/min; nebulizer at 35 PSI; Vcap at 3500 V; Fragmentor at 175 V. Spectra were collected at 1/sec (13,701 transients/spectrum) in the 85–1,200 m/z range. Reference mass solution (121.050873 m/z and 922.009798 m/z) was infused constantly via a second nebulizer at 15 psi. Cysteine (cys) and cystine (cys^2^) were measured by mining the data for the appropriate cys and cys^2^ masses ([M + H]^+^ signals at 122.0248 and 241.0283 m/z, respectively). l-Trp-_*d5*_ (exact mass 209.1213; detected as [M + H]^+^ at 210.1285 m/z) was used as an internal standard. Peak areas were obtained using Agilent Mass Hunter Qualitative Analysis software (VB05) (Agilent Technologies), and converted to absolute amounts using calibration curves created with authentic cys (LOD < 1 nmol/mL) and cys^2^ (LOD < 0.5 nmol/mL).

### Cyanide quantification

The petioles of 5-week-old Col-0, *cyp79b2 cyp79b3*, and *cyp71a12 cyp71a13* leaves were cut and submerged in 10 mL of water contained in a small Petri plate covered with parafilm. Four leaves were used in the preparation of one biological replication (Petri dish). One biological replication per plant genotype per treatment (± mites) per trial was collected in five independent trials. Each set of four leaves was infested with ∼2,000 bean-a mites (prestarved overnight) and closed with a vented lid to confine mites. After 2 h of infestation mites were removed from leaves with a vacuum. Leaves were quickly weighed and frozen in liquid N_2_. Approximately 200 mg of the frozen leaf tissue was homogenized in 1.5-mL Eppendorf tubes twice for 2 min at maximum speed within a Retsch ball mill (MM400; Retsch, Haan, Germany). Metabolites were extracted in 200 µL of ammonium acetate 10 mM for 10 min in a thermal mixer at 25°C and 600 rpm. Samples were centrifuged 15 min at 15,000 rpm at 4°C, and free HCN was derivatized by adding 50 µL of sample, 50 µL of 10 mM ammonium acetate (pH 7.5), 50 µL of 4 mM 2,3-naphthalenedialdehyde and 50 µL of 50 mM taurine to produce a N-substituted 1-cyano[f]benzoisoindole (CBI) (Garcia et al., 2010). The mixed samples were incubated in the dark for 15 min. After derivatization, the samples were centrifuged for 5 min at room temperature at 15,000 rpm and analyzed by HPLC-MS/MS. Derivatized CBI samples were analyzed using an ExionLC AD HPLC (Sciex, Framingham, MA, USA) with a reverse phase column Kinetex XB-C18 RP column (100 × 4.6 mm, 2.6 μm particle size, 100 Å pore size) protected by a Synergi 2.5 μ Fusion-RP 100 Å C18 (both Phenomenex, Torrance, CA, USA) guard cartridge (10 Å∼2.00 mm, i.d.). Each chromatographic analysis was carried out with mobile phase components of aqueous 10 mM ammonium acetate (mobile phase A) and 10 mM ammonium acetate in methanol (mobile phase B). An aliquot (10 μL) of the prepared sample was separated by gradient flow at 250 µL min^−1^ and 40°C. The concentration of B, initially 50%, was increased linearly to 100% over 3 min, held at 100% for 1 min, decreased linearly to 50% over 1 min, and held constant for 2 min to re-equilibrate the column between samples. A Sciex QTRAP 6500+ MS-MS (Applied Biosystems, Foster City, CA, USA) equipped with an electrospray ionization source operating in negative ionization mode using an ion spray voltage of −4500 V. Further ESI parameters were: curtain gas, 30 psi; low-pressure collision gas; temperature, 500°C; nebulizer gas (GS1), 40 psi; heater gas (GS2), 60 psi. Data were acquired with Analyst version 1.7 software in multiple reaction monitoring mode with a detection window of 60 s. The ionization adducts measured [M-H]^−^ selected for identification and quantification were 298.6 m/z (Q1) and 191.0 m/z (Q3). Declustering potential of −35.0 V and collision energy of −30.0 V were used. Data were processed with Sciex OS software for peak integration and quantification. For quantification, an external standard curve with NDA/taurine derivative of a commercial cyanide standard solution was used. Statistical analysis was performed using a two-way ANOVA with experimental trial (blocking factor), and mite treatment as main factors without an interaction term for the experiment on Col-0. When more than one plant genotype was being assessed, a three-way ANOVA was used with experimental trial (blocking factor), mite treatment and plant genotype as main factors with an interaction term between mite treatment and plant genotype included (Brady et al., 2015) followed by Tukey’s HSD test upon detection of a significant interaction.

## Accession numbers

Sequencing data used in this study were deposited to NCBI SRA under BioProject PRJNA701185.

## Supplemental data

The following materials are available in the online version of this article.

**Supplemental Table S1.** List of *T. urticae* genes associated with mite adaptation to CYP79B2 CYP79B3-derived Arabidopsis defenses.

**Supplemental Table S2.** List of primers used in this study.

## Funding

This work was supported by the Government of Canada through the Ontario Research Fund (RE08-067) awarded to M.G. and V.G. and the Natural Sciences and Engineering Research Council of Canada (NSERC, RGPIN-2018-04538) awarded to V.G. This work was partially funded by USDA’s National Institute of Food and Agriculture, award # 2020-67014-31179 through the NSF/NIFA Plant Biotic Interactions Program awarded to M.C., M.G., and V.G.

*Conflict of interest statement*. The authors declare that they have no conflicts of interest with the contents of this article.

## Supplementary Material

kiac147_Supplementary_Data_Tables_S12

## Notes

V.G. and S.D. conceived the research plans. V.G. supervised the experiments. S.D. with the help of E.W., N.B., and A.S. performed all experiments testing the requirement of TuCAS. G.S. prepared samples for RNASeq analysis that was performed by V.Z. K.B. and L.C.R. prepared samples and analyzed cyanide levels in Arabidopsis plants. M.M. and N.B. performed in situ localization of TuCAS. M.B. analyzed cysteine levels. S.D., V.Z., K.B., M.A.B., and V.G. analyzed the data. All authors contributed to the preparation of the manuscript. L.C.R., M.C., M.A.B., M.G., and V.G. contributed resources and equipment. V.G. is the author responsible for contact and communication.

The author responsible for distribution of materials integral to the findings presented in this article in accordance with the policy described in the Instructions for Authors (https://academic.oup.com/plphys/pages/general-instructions) is: Vojislava Grbić (vgrbic@uwo.ca).
