# Supplementary material for: β-Cyanoalanine synthase protects mites against Arabidopsis defenses
Source: Plant Physiol. 2022 Mar 28;189(4):1961–75. doi: 10.1093/plphys/kiac147 (PMC9342966; doi:10.1093/plphys/kiac147)
Supplement: kiac147_Supplementary_Data_Tables_S12 [file kiac147_supplementary_data_tables_s12.pdf]

Supplemental Table S1. List of *Tetranychus urticae* genes associated with mite adaptation to CYP79B2/CYP79B3-dependent Arabidopsis defenses.

ID	Symbol	Description	GO	LFC.Mite			F	P.Value	adj.P.Val
				Col.v.Lnd	Cyp.v.Lnd	Col.v.Cyp			
tetur02g00510	-	neprilysin	GO:0006508<br>GO:0004222<br>GO:0004222<br>GO:0006508<br>GO:0008237	2.920	0.344	2.575	70.361	2.338E-11	1.399E-09
tetur02g02590	-	serine/threonine-protein phosphatase 6 regulatory subunit 1	GO:0005488<br>GO:0005488	2.489	0.802	1.688	55.762	3.006E-10	1.355E-08
tetur02g05270	-	Hypothetical protein	-	1.598	0.213	1.385	12.897	1.210E-04	1.125E-03
tetur02g07220	-	Galactose-binding domain-like	-	2.402	0.057	2.345	43.985	3.624E-09	1.231E-07
tetur02g90622	-	hypothetical protein	-	2.355	0.486	1.869	20.294	4.416E-06	6.345E-05
tetur03g08460	-	Hypothetical protein	-	2.682	0.125	2.557	13.160	1.059E-04	1.010E-03
tetur05g02210	RNI	Leucine-rich repeat domain, L domain-like	GO:0005515<br>GO:0005515<br>GO:0005515	2.164	0.682	1.482	10.190	5.166E-04	3.908E-03
tetur05g05300	TuGSTm12	Glutathione S-transferase; class mu	GO:0005515<br>GO:0004364<br>GO:0005515<br>GO:0008152	1.486	0.240	1.247	35.832	2.799E-08	7.449E-07
tetur07g02230	RNI	Leucine-rich repeat domain, L domain-like	GO:0005515<br>GO:0005515<br>GO:0005515	1.386	-0.439	1.825	25.206	7.180E-07	1.306E-05
tetur07g03820	BolA-2	BolA-like protein	-	1.286	0.156	1.130	96.569	6.204E-13	5.633E-11
tetur07g03950	-	acylamino-acid-releasing enzyme	GO:0006508<br>GO:0008236	2.571	0.685	1.886	192.997	1.408E-16	5.149E-14
tetur07g05780	-	Scramblase	-	1.382	0.348	1.034	72.611	1.642E-11	1.051E-09
tetur07g05860	-	phospholipid scramblase 2	-	3.609	0.566	3.043	45.705	2.444E-09	8.642E-08
tetur07g06390	UGT36	UDP-glycosyltransferase; teturUGT36	GO:0008152<br>GO:0016758<br>GO:0008152<br>GO:0016758	2.046	0.404	1.641	33.749	4.983E-08	1.232E-06
tetur08g00010	-	Fibroin (partial)	-	1.028	0.023	1.005	40.159	9.096E-09	2.786E-07
tetur08g08369	-	hypothetical protein	-	1.508	0.467	1.041	58.010	1.962E-10	9.268E-09
tetur09g00350	TuPap-32	Cathepsin L	GO:0008234<br>GO:0006508<br>GO:0006508<br>GO:0008234	1.473	-0.392	1.865	35.623	2.962E-08	7.860E-07

tetur09g06716	ADAMX3	Peptidase M12B, ADAM/reprolysin	GO:0004222 GO:0006508 GO:0008237 GO:0004222 GO:0006508 GO:0008237 GO:0004222 GO:0006508 GO:0008237	2.674	0.977	1.696	43.702	3.870E-09	1.301E-07
tetur101g00010	-	intraflagellar transport protein 81 homolog	GO:0030992 GO:0042384 GO:0015631 GO:0042073 GO:0042384 GO:0030992 GO:0015631 GO:0042073 GO:0042384 GO:0030992 GO:0015631 GO:0042073 GO:0015631 GO:0030992 GO:0042073 GO:0042384	2.798	0.402	2.396	140.432	7.074E-15	1.132E-12
tetur106g00050	-	PREDICTED: similar to Y41C4A.8	-	1.354	0.244	1.109	63.660	7.107E-11	3.714E-09
tetur10g01570	CAS	cysteine synthase A	GO:0003824 GO:0008152 GO:0030170	1.796	0.505	1.291	9.893	6.121E-04	4.522E-03
tetur10g02060	-	glutamine-dependent NAD	-	1.800	0.118	1.682	30.303	1.372E-07	3.017E-06
tetur11g00970	-	PREDICTED: similar to limkain b1	GO:0005777 GO:0010468	1.972	0.659	1.313	122.612	3.650E-14	4.537E-12
tetur11g01790	-	WD40/YVTN repeat-like-containing domain	GO:0005515 GO:0005515	2.950	0.996	1.954	258.983	3.493E-18	2.353E-15
tetur11g04600	-	tRNA	-	1.779	0.343	1.436	25.461	6.575E-07	1.209E-05
tetur11g05200	TuABCA-05	ABC-transporter; class A	GO:0005524 GO:0016887 GO:0005215 GO:0005524 GO:0006810 GO:0016021 GO:0016887	1.891	0.318	1.573	16.503	2.175E-05	2.578E-04
tetur12g02750	-	Hypothetical protein	-	3.372	0.091	3.282	26.107	5.277E-07	9.906E-06

tetur12g02810	ovo	ovo	GO:0008270<br>GO:0005622<br>GO:0003676<br>GO:0000981<br>GO:0003676<br>GO:0003700<br>GO:0005622<br>GO:0005634<br>GO:0006351<br>GO:0006366<br>GO:0008270<br>GO:0046872	2.042	0.688	1.354	114.392	8.378E-14	9.492E-12
tetur12g03770	-	PREDICTED: alpha 1 type XIII collagen	-	1.038	0.019	1.019	85.813	2.447E-12	1.887E-10
tetur14g92990	-	hypothetical protein	-	2.057	0.872	1.185	19.361	6.426E-06	8.778E-05
tetur171g90421	-	hypothetical protein	-	1.783	0.587	1.196	92.812	9.861E-13	8.305E-11
tetur17g03650	-	Major facilitator superfamily domain	-	1.409	0.273	1.136	23.747	1.201E-06	2.025E-05
tetur20g02620	-	Galactose-binding domain-like	-	3.456	0.675	2.781	112.314	1.042E-13	1.160E-11
tetur214g00010	-	GPCR, family 2, secretin-like	GO:0004888<br>GO:0004930<br>GO:0007166<br>GO:0007186<br>GO:0016020<br>GO:0016021<br>GO:0004888<br>GO:0004930<br>GO:0007166<br>GO:0007186<br>GO:0016020<br>GO:0016021	1.976	0.326	1.649	20.950	3.414E-06	5.059E-05
tetur21g01400	UGT58p	UDP-glycosyltransferase; teturUGT58p	GO:0008152<br>GO:0016758<br>GO:0008152<br>GO:0016758	2.951	0.988	1.963	12.657	1.368E-04	1.254E-03
tetur21g01830	-	Hypothetical protein	-	1.679	0.240	1.440	22.409	1.959E-06	3.081E-05
tetur21g03100	CuffL6	Cutoff-like protein	-	2.385	0.448	1.937	20.426	4.191E-06	6.063E-05
tetur23g00870	-	WD and tetratricopeptide repeats protein 1	GO:0005515<br>GO:0005515<br>GO:0005515	1.560	0.421	1.139	40.545	8.265E-09	2.562E-07
tetur23g91451	-	hypothetical protein	-	1.070	-0.177	1.247	22.338	2.012E-06	3.149E-05

tetur247g00010	-	ATG4 autophagy related 4 homolog A	-	1.411	0.157	1.255	175.302	4.648E-16	1.293E-13
tetur24g02120	-	papillary renal cell carcinoma	-	2.276	-0.225	2.501	16.136	2.564E-05	2.957E-04
tetur25g01650	-	Dynamin GTPase effector	GO:0003924 GO:0005525 GO:0003924 GO:0005525	1.836	0.819	1.017	65.401	5.276E-11	2.838E-09
tetur26g01210	-	Hypothetical protein	-	2.174	0.213	1.960	11.509	2.497E-04	2.113E-03
tetur26g01370	AKD1A	Ankyrin repeat-containing domain	GO:0005515 GO:0007165 GO:0005515 GO:0007165 GO:0005515 GO:0007165	1.254	0.206	1.048	111.552	1.131E-13	1.227E-11
tetur26g01790	-	nucleoside diphosphate-linked moiety X motif 19; mitochondrial precursor	GO:0016787 GO:0016787	1.369	0.212	1.158	34.947	3.565E-08	9.183E-07
tetur30g01120	-	Plexin domain-containing protein	-	1.784	0.413	1.372	33.059	6.066E-08	1.457E-06
tetur315g90211	-	hypothetical protein	-	1.918	0.315	1.602	24.281	9.924E-07	1.717E-05
tetur31g01520	-	intraflagellar transport protein 81 homolog	GO:0015631 GO:0030992 GO:0042073 GO:0042384 GO:0042384 GO:0030992 GO:0015631 GO:0042073 GO:0015631 GO:0030992 GO:0042073 GO:0042384	1.495	-0.340	1.834	71.838	1.852E-11	1.151E-09
tetur32g00670	-	SIT4 phosphatase-associated protein	GO:0005488 GO:0005488	2.325	0.702	1.623	31.045	1.096E-07	2.457E-06
tetur32g02180	CBR1p	Carbonyl reductase [NADPH] 1; pseudo gene	GO:0008152 GO:0016491 GO:0008152 GO:0016491	1.842	0.715	1.127	13.411	9.342E-05	9.026E-04
tetur34g00170	RNI	Leucine-rich repeat domain, L domain-like	GO:0005515 GO:0005515	1.995	0.749	1.245	69.950	2.496E-11	1.473E-09
tetur34g00180	RNI	Leucine-rich repeat domain, L domain-like	GO:0005515 GO:0005515 GO:0005515	1.922	0.481	1.441	9.687	6.894E-04	4.995E-03

tetur34g00300	RNI	Leucine-rich repeat domain, L domain-like	GO:0005515<br>GO:0005515<br>GO:0005515	1.359	0.239	1.120	18.832	7.986E-06	1.063E-04
tetur352g00020	-	Hypothetical protein	-	1.150	0.143	1.007	14.496	5.499E-05	5.724E-04
tetur352g00040	-	Hypothetical protein	-	1.238	-0.105	1.342	25.441	6.621E-07	1.216E-05
tetur392g00010	-	Hypothetical protein	-	2.506	0.872	1.635	161.199	1.309E-15	2.659E-13
tetur49g00020	RNI	Leucine-rich repeat domain, L domain-like	GO:0005515<br>GO:0005515	2.092	-0.289	2.381	55.503	3.160E-10	1.405E-08
tetur640g00020	-	trans-1;2-dihydrobenzene-1;2-diol dehydrogenase	GO:0003824<br>GO:0005488<br>GO:0008152<br>GO:0016491<br>GO:0003824<br>GO:0005488<br>GO:0008152<br>GO:0016491<br>GO:0003824<br>GO:0005488<br>GO:0008152<br>GO:0016491	1.700	-0.084	1.784	66.514	4.376E-11	2.436E-09
tetur82g00020	RNI	Leucine-rich repeat domain, L domain-like	GO:0005515<br>GO:0005515<br>GO:0005515<br>GO:0005515<br>GO:0005515<br>GO:0005515	1.127	-0.071	1.198	76.573	9.007E-12	6.037E-10

Supplemental Table S2. List of primers used in this study.

Name	Application	Forward primer sequence (5'-3')	Reverse primer sequence (5'-3')	Efficiency
TuCAS-T7	RNAi	[T7]- ATGACTGAGTCAACTGTCGAC	[T7]- AACAAAACAGCCTCCAGTTCC	NA
TuCAS-RT	RT-qPCR	ACCATCACCAAGCCTCTTCA	TGCTTCTTCGTCACTTACAGAG	99.8%
TuCAS-1-T7	RNAi	[T7]- GGAACTTCCAAATTTTTAAAAGAA	[T7]- GCAATCAGATTTAGATTTTCACC	NA
TuCAS-1-RT	RT-qPCR	CTGGACCAGGACGACTTTTG	CCATCCTTGAGTTGGCCTGA	99.2%
RP49	RT-qPCR	CTTCAAGCGGCATCAGAGC	CGCATCTGACCCTTGAACTTC	100.9 %
TuCAS-ISH sense	*in situ*	[T7]- ATGACTGAGTCAACTGTCGAC	AACAAAACAGCCTCCAGTTCC	NA
TuCAS-ISH antisense	*in situ*	ATGACTGAGTCAACTGTCGAC	[T7]- AACAAAACAGCCTCCAGTTCC	NA
